# Impact of Poultry Farmers’ Participation in Modern Food Retail Markets on Household Dietary Diversity: Lessons from Southeast Nigeria

**DOI:** 10.3390/ani10040611

**Published:** 2020-04-02

**Authors:** Robert Ugochukwu Onyeneke, Chukwuemeka Chinonso Emenekwe, Nneka Maris Chidiebere-Mark, Jane Onuabuchi Munonye, Jonathan Ogbeni Aligbe, Clementina Kanu, Chibuzo Uzoma Izuogu, Chukwudi Loveday Njoku, Uwazie Iyke Uwazie, Christian Obioma Uwadoka, Gillian Chidozie Azuamairo

**Affiliations:** 1 Department of Agriculture (Agricultural Economics and Extension Programme), Alex Ekwueme Federal University Ndufu-Alike, Ebonyi State 482131, Nigeria; munojane@gmail.com (J.O.M.); chibuzoizuogu@gmail.com (C.U.I.); chukzy4now1980@gmail.com (C.L.N.); 2 Department of Economics and Development Studies, Alex Ekwueme Federal University Ndufu-Alike, Ebonyi State 48213, Nigeria; emenekwe.c@gmail.com; 3 Department of Agricultural Economics, Extension and Rural Development, Imo State University Owerri, Owerri 460222, Nigeria; nnekachidieberemark@yahoo.com; 4 Department of Planning and Policy Coordination, Federal Ministry of Agriculture and Rural Development, Benin City 300251, Nigeria; jonathan.aligbe@gmail.com; 5 Department of Accountancy/Banking and Finance, Alex Ekwueme Federal University Ndufu-Alike, Ebonyi State 482131, Nigeria; srmenfu2009@gmail.com; 6 Department of Economics, Michael Okpara University of Agriculture Umudike, Abia State 440109, Nigeria; ui.uwazie@yahoo.com; 7 Centre for Partnerships, Alex Ekwueme Federal University Ndufu-Alike, Ebonyi State 482131, Nigeria; chrisuwadoka@hotmail.com; 8 Department of Agriculture (Agribusiness and Management Programme), Alex Ekwueme Federal University Ndufu-Alike, Ebonyi State 482131, Nigeria; ebufaith@gmail.com

**Keywords:** modern food retail markets (MFRM), dietary diversity, poultry farm household, FANTA, instrumental variable regression, seemingly unrelated regression

## Abstract

**Simple Summary:**

Developing countries’ food systems are experiencing rapid transformations led by modern food retail market (MFRM) channels such as supermarkets, fast food firms, hotels, and convenience stores. This paper analyzes the impact of these channels on farm households’ dietary diversity with survey data from Southeast Nigeria. Estimates from the instrumental variable model show that participation in MFRM is associated with a significant increase in dietary diversity. Furthermore, the linkages through which MFRM participation impacts dietary diversity are analyzed using seemingly unrelated regression. Poultry farm income, consumption of poultry products produced by the farmer, and area of vegetable cultivated using poultry droppings have positive association with dietary diversity, while male controlled poultry farm revenue has negative association with dietary quality. Our study provides useful insights that poultry farm managers would find helpful. It also serves as a potential source of information for policymakers for planning as it links smallholder poultry farmers’ participation in modern food retail markets to improved nutrition.

**Abstract:**

This study analyzed the interrelationships between participation in MFRMs and dietary diversity of poultry farming households in Southeast Nigeria. We used cross-sectional data from poultry farmers in Southeast Nigeria and employed instrumental variable and seemingly unrelated regression models to estimate the impact of MFRM participation and major linkages to poultry farm households’ dietary diversity. The results show that participating in MFRMs, relative to traditional markets, improved poultry farmers’ dietary diversity. Moreover, dietary diversity was positively related to higher poultry farm incomes, higher value of own poultry products consumed, and larger area of vegetable cultivated using poultry droppings as manure. Furthermore, increased poultry farm income, higher value of own poultry products consumed, and larger area of vegetable land cultivated using poultry droppings as manure increased the dietary diversity of the farm households. In contrast, a higher share of poultry production revenue controlled by men reduced household dietary diversity. These findings make clear the potential of improving farming households’ nutrition outcomes by promoting participation in MFRMs and the major impact pathways.

## 1. Introduction

Developing countries’ food systems are experiencing rapid transformation and modernization. Modern food retail markets (MFRM) (such as supermarkets, hotels, fast food firms, and convenience stores) are rapidly replacing traditional markets [1,2]. This transformation and modernization are driven by demand and supply. On the demand side, factors such as high rate of urbanization, increasing incomes and lifestyles potentially pull the consumer towards consumption of high value foods (including processed foods), which are better provided by modern retail channels than the traditional channels [3,4]. Supply side factors are fostered by policy preferences such as the liberalization of the food sector, which enables the provision of diverse products and quality to consumers. Supply side factors have also led to value chain improvements. MFRM channels establish supply agreements with farmers, with the potential to improve farmers’ welfare especially nutrition [5,6].

The existing literature have analyzed the relationship between MFRM channels participation and labor markets [7,8,9], agricultural productivity [5,7], and farming household income [1,5,10]. These studies mainly find that MFRM channels can boost the rural farm sector and rural economic growth. Furthermore, the rapid pace of food retail modernization is considered a major factor in the nutrition transition and security of most developing countries [11]. The net impact of the food market modernization on the nutrition of different segments of the population of developing countries, especially sub-Saharan African countries, is yet unclear. Although, there are studies on the impacts of food market modernization on nutrition and diet quality of households in developing countries [4,12,13,14,15,16,17,18,19,20,21], but the evidences produced by the studies are mixed. For example, some of the studies show that access to MFRM increases consumption of unhealthy processed foods [4,12,13,14,15,16,17,18,19]. Conversely, Tessier [20] found an increase in dietary quality among Tunisian households that regularly visit supermarkets. Moreover, Rupa et al. [21] found positive effects of modern food market expenditure on consumption of iron, protein, and dietary diversity in Vietnam.

More so, the literature on impacts of farmers’ participation in MFRM channels in Africa is growing see [4,5,7,10,22,23,24,25]. These studies [4,5,7,10,22,23,24,25], have focused mainly on the participation of vegetable farmers in MFRM. Studies on the impact of participation in MFRM on poultry farm households’ dietary diversity in Africa are rare. Furthermore, studies on farmers’ participation in MFRMs in Nigeria, Africa’s most populous country and a nation with rapidly increasing MFRMs and urban population, are almost nonexistent. This study, therefore, assessed MFRMs participation and its effects on poultry farm households’ dietary diversity.

Nigeria presents an interesting case; it has a population of more than 190 million persons, which accounts for about 15.19% of Africa’s population, with about half of this population living in urban areas [26,27]. Rapid urban population growth, a growing middle class with corresponding increasing disposable income and the drive by government to modernize retailing have led to changing consumption patterns and increasing demand for processed and easy-to-cook foods such as poultry, meat, and egg. Shopping in decent and organized outlets and buying neatly-arranged and labeled poultry products are the experiences of Nigerian urban consumers [26]. Supermarkets, convenience stores, hotels, and fast-food firms are better placed to provide such products and services than the traditional markets. Policies supporting diversification of the economy and greater involvement of private sector operators in the food industry have created an enabling environment for the operations of supermarkets, convenience stores, hotels, and fast food firms, described as MFRMs in this study. Consistency in offering good-quality products to their customers is one of the main marketing strategies of the MFRMs [1]. To achieve this, MFRMs are changing their sourcing and procurement systems and policies to involve centralized buying points and enter into a supply agreement with farmers and traders [1,24].

Nigeria has the largest market for poultry products in Africa because the poultry sector contributes about ₦80 billion ($600 million) to the economy of Nigeria and the size of the sector is approximately 165 million birds, which produces about 0.65 million metric tonnes of eggs and 0.29 million metric tonnes of poultry meat [28]. The local/traditional chickens constitute the majority (about 84.2%) of Nigeria’s poultry population thus, providing the largest share of poultry meat in Nigeria [29,30]. Production is classified into three sectors, following the FAO, as follows: The industrial sector (this includes highly integrated industries with high-level of automation), the commercial sector (this includes all minimum-to-large scale commercial poultry farmers producing), and traditional sector (this includes all forms of traditional scavenging or free-range birds) [30]. The bulk of the poultry products of these sectors are consumed by urban dwellers, who make purchases from supermarkets, convenience stores, hotels, and fast-food firms.

There are several pathways through which participation in MFRM chains affects farming households’ nutrition [1]. This paper hypothesizes that poultry farmers’ participation in MFRM is both directly and indirectly linked with their dietary diversity. Chege et al. [1] identified three linkages through which MFRM participation affects household nutrition. These include (i) household income: Increasing incomes enables farmers to purchase more food; (ii) production choices: This increases specialization and can increase the availability of home-produced foods; and (iii) gender roles: This leads to better food sales and purchase decisions within the household. Our study followed these three pathways identified by Chege et al. [1] and added a fourth one—using poultry droppings for cultivation of nutritious vegetable crops.

Participation in MFRM channels affects household nutrition of poultry farmers through income changes, which increases farmers’ access to food and diversified diets [1,10,22]. According to Rao and Qaim [22], involvement in MFRM channels can increase farmers’ incomes and may lead to increased consumption of diversified diets through purchase of more diverse foods. The second is through consumption of poultry products produced by the farmers. Poultry farmers, who supply MFRMs, are usually large farmers and may have more poultry products at their disposal for consumption. The third linkage is through the production of vegetables (for sale and consumption) using poultry dropping as manure. The market for vegetables is increasing in Nigeria and farmers who, hitherto, do not produce vegetables are diversifying into vegetable production [31]. Poultry farmers are at an advantaged position because of the increasing demand for poultry dropping as manure for such business. It is expected that poultry farmers who supply MFRMs have large farms and should have more quantity of poultry dropping for their vegetable production. The fourth pathway is through changing gender roles within the household. Small-scale poultry production is mainly carried out by women in Nigeria mainly for household consumption [32,33,34]. With increasing commercialization of poultry business, men are becoming more involved in poultry production and this is expected to affect the intra-household gender decisions on control of poultry farm revenue, as well as food consumption and dietary diversity [25].

## 2. Materials and Methods

### 2.1. Sampling Procedure and Data Collection

Between December 2018 and June 2019, we conducted a cross-sectional survey of poultry farmers in Southeast Nigeria. Southeast Nigeria comprises five states—Abia, Anambra, Ebonyi, Enugu, and Imo States. Some poultry farmers in this part of Nigeria engage in production of poultry products such as egg and meat. Some supply to MFRMs while many others sell in the traditional markets. Two states out of the five states were chosen for this study based on the preponderance of supermarkets, convenience stores, hotels, restaurants, and fast foods. In this study, we define these markets and firms as modern food retail markets. Based on information from agricultural extension agents in the selected states, five major areas of poultry production in each state were selected. From these areas, poultry farmers were selected. Overall, two hundred and twenty (220) commercial and traditional sector poultry farmers were sampled—60 engaged in MFRM channels and 160 participated only in traditional market channels.

Food frequency data, socioeconomic characteristics, poultry production and marketing data, and other economic activities data were collected from the sampled poultry farmers with the aid of a structured and carefully designed questionnaire. The authors obtained consents from the sampled poultry farmers after informing them the purpose of the research and assuring them absolute confidentiality of any information they would provide. To further guarantee the surveyed farmers absolute confidentiality for all information they would provide, the authors anonymized the questionnaire. The authors also made it known to the farmers that their participation in the survey was voluntary. Poultry farming household heads and/or their spouses were interviewed. A seven-day dietary recall was included in the interview. The person in-charge of cooking and food choices, if different from the household head, was interviewed together with the household head. Food frequency data was collected for all foods consumed in the household and the data was used to compute the dietary diversity of each household as a measure for nutrition. This index—dietary diversity score—is a metric for measuring nutritional quality of households and individuals [35,36,37] and impacts of nutrition interventions [38].

### 2.2. Household Dietary Diversity Indicator

We adopted the Food and Nutrition Technical Assistance (FANTA) guide in computing the dietary diversity score of households. This guide classifies foods consumed by human beings into twelve broad categories which is a reflection of nutrition and dietary quality [35,39]. The various classes of food according to FANTA are “cereals/grains, fish and seafood, root/tubers and plantain, seeds/pulses/nuts, vitamin A-rich fruits and vegetables, other fruits and vegetables, milk and milk products, oil/fats, meat (organ and flesh meat) and edible insects, sugar/honey, eggs, miscellaneous (spices, condiments and beverages)” [39,40]. This implies that the HDDS yields a value between zero (for households that consumed no food) and twelve (households that consumed foods in all the groups) for each day. We added the daily dietary diversity score of each household and divided the sum by seven to represent the dietary diversity score for the seven-day dietary recall conducted. This implies that we used the average dietary diversity score of the seven-day period as the outcome variable of this study. Onyeneke et al. [35] highlighted that one of the advantages of using the FANTA guideline to measure dietary diversity and nutritional quality is its elaborateness and robustness in clearly capturing foods rich in micronutrients (vitamin A and other minerals) separately to check the existence of hidden hunger in the households.

### 2.3. Econometric Framework

For an unbiased estimation of participation in MFRMs and its effects on dietary diversity of poultry farmers, we adopted the instrumental variable regression model. One of the greatest problems of impact estimation is bias and this is controlled by choosing suitable/appropriate econometric tools. Poultry farmers self-select themselves to participate in MFRMs channels. This makes the treatment endogenous and addressing the endogeneity of the treatment variable needs the identification of an instrument variable. A suitable instrument must not have any relationship with the outcome variables/dependent variables (exclusion criterion), but must be significantly related to the endogenous variable (relevance criterion) [41,42]. The instrument identified in this study is in contact with agricultural extension agents. This variable fulfilled the two criteria for choosing suitable instruments. The variable is significantly correlated with MFRMs participation. The variable also demonstrated no significant relationship with dietary diversity in the simple regression model the authors conducted between the contact with agricultural extension and dietary diversity. Agricultural extension service is becoming more market-oriented and farmers need information about emerging market opportunities. In Nigeria, the increasing demand for easy-to-cook agricultural products by consumers is driving the growth experienced in MFRMs. This change and preference for easy-to-cook products drives uptake of innovations in marketing farmers’ products rather than technologies [43,44]. It is, therefore, valid to have extension contact significantly affect farmers’ participation in MFRMs. Agricultural extension services showed no relationship with the outcome/dependent variable (dietary diversity) but would only affect it through participation in MFRMs channel.

The instrumental variable model used in estimating the impact of MFRM participation on dietary diversity of poultry farm households is stated thus:

(1) $$Y=\delta_{0}+\delta_{1}P+\delta_{2}X_{1}+\varepsilon_{1}$$

(2) $$P=\beta_{1}T+\beta_{2}X_{1}+\varepsilon_{2}$$

where:

Y = dietary diversity score.

P = participation in MFRM (dummy variable; 1 = participated, 0 = otherwise).

X_1_ = vector of control variables (such as age, marital status, household size, gender, education, poultry farming experience, distance to the market, membership of cooperative society/farmer association, access to credit, other socioeconomic and institutional characteristics, and ownership of household assets) that may influence HDDS.

T = instrument variable.

ε_1_ and ε_2_ = error terms.

Seemingly unrelated regression was used to test the hypothesis of the impact pathways as stated below:

(3) $$Y=\delta_{0}+\delta_{1}I+\delta_{2}W+\delta_{3}V+\delta_{4}S+\delta_{4}X_{2}+\varepsilon_{3}$$

(4) $$I=\sigma_{0}+\sigma_{1}P+\sigma_{2}X_{3}+\varepsilon_{4}$$

(5) $$W=\theta_{0}+\theta_{1}P+\theta_{1}X_{4}+\varepsilon_{5}$$

(6) $$V=\pi_{0}+\pi_{1}P+\pi_{2}X_{5}+\varepsilon_{6}$$

(7) $$S=\alpha_{0}+\alpha_{1}P+\alpha_{0}X_{6}+\varepsilon_{7}$$

where:

Y = dietary diversity, which is a function of household income (I), value of own poultry products consumed (W), size of vegetable farm/garden cultivated using poultry droppings (V), gender of the person in the household who is in-charge of poultry revenues (S), (X_2_) = vector of control variables which are mainly socioeconomic factors. From the foregoing, I, W, V, and S are influenced by participation in MFRM supply channels (P), and additional covariates (X_3_ to X_6_).

## 3. Results

### 3.1. Socioeconomic Characteristics

Table 1 reports the descriptive statistics of the socioeconomic characteristics of the sampled households. The average ages of the household heads and their spouses were 46.93 and 41.37 years, respectively. The average household size in the sample was five members and they depended on poultry farming for their livelihood. About 27% of the poultry farmers participated in MFRMs supply arrangement, with an average poultry farming experience of 11.94 years.

The poultry farmers cultivated an average of 0.18 ha of land with vegetables using poultry dropping as manure. The average income from poultry production was ₦787,054.50 per annum. It takes 37.58 min on average to get to the market on foot, and costs ₦159.25 to transport poultry products to the market. Men control about 53% of the revenue from poultry production. Own consumption by the poultry farming households was ₦49,923.54 per annum.

Fifty-six percent and thirty-four percent of the farmers had access to veterinary and extension services, respectively. Eighteen percent of the farmers had access to credit, 59% had refrigerator access, 70% had access to electricity. Sixty-five percent owned a television set, 83% had radio, while 90% owned a mobile phone. Thirty-five percent of the farmers owned a car, while about half of household heads belonged to a cooperative society and earn about ₦51,964 from off-farm incomes.

### 3.2. Household Dietary Diversity

Table 2 reports the dietary diversity score of the poultry farming households. We disaggregated the results into three categories to reflect the participation in different market channels. These categories are poultry farmers engaged in MFRM, their counterparts engaged in the traditional market channels, and the whole sample. For households involved in MFRMs, the average dietary diversity score was 8.25; this implies that participating households consumed an average of eight food groups. For households using the traditional market channel, the average dietary diversity score was 5.74, which is lower than those using the MFRM channel. The whole (or pooled) sample dietary diversity score was 6.43.

### 3.3. Econometric Results

#### 3.3.1. Factors influencing MFRM participation: Instrumental variable analysis

Table 3 reports instrumental variable regression results of the predictors of MFRM participation. The result shows that the instrument, extension contact, was significant and positively linked with participation in MFRM. Extension workers can provide the farmers with information about markets and opportunities. The coefficient of gender, marital status, and number of under-five children were positive and significant, implying that married male poultry farmers with higher number of under-five children would have greater likelihood to participate in MFRM than their unmarried female counterparts who have less responsibility and dependents to take care of. Years of poultry farming experience, education of the sampled farming household heads and their spouses had a positive impact on MFRM participation. This implies that higher educational attainment and experience may enable poultry farmers to enter into supply contracts with MFRM and consequently sell their products through MFRM channels.

The coefficients of ownership of information and communication media such as radio and mobile phone were significant and positive, implying that media programming for the public fosters greater engagement in MFRM supply channels. The coefficient of car ownership was positive and significant, implying that farmers who own cars will use cars more than those farmers serving traditional markets. Improved access to credit and veterinary services had significant and positive coefficients. The results also showed that household head’s and spouse’s membership of cooperative society had positive and significant impacts on MFRM participation.

The results show that the coefficients of infrastructural variables such as access to electricity, ownership of refrigerator, and travel time to nearest markets had positive relationships with participation in MFRM. Finally, the coefficient of off-farm income was positive and significant. This implies that farming households with higher off-farm income participated more in MFRM than their counterparts with lower off-farm income.

#### 3.3.2. Factors influencing dietary diversity

Table 3 (column 2) reports the second stage of the instrumental variable regression with dietary diversity score as the dependent variable. The coefficient of MFRM participation was significantly and positively related to dietary diversity. This implies that participation in MFRM channels significantly increased household dietary diversity score.

In terms of control variables, male-headed household, being married, presence of young children and infants (children year years and younger) and number of women in reproductive age in the household were found to significantly increase dietary diversity. Spouse’s age significantly and negatively impacted dietary diversity indicating that farmers having younger spouses ate more diversified diets/foods in their families than their counterparts whose spouses are older. Higher educational attainment of the household heads and their spouses significantly increased household dietary diversity. Furthermore, access to electricity, access to means of communication, membership of farmer groups significantly increased poultry households’ dietary diversity. The latter result is expected, since membership of such groups makes it possible to obtain new information on best practices, as well as ease of access to improved varieties of poultry animals. Off-farm employment and income increased dietary diversity. Since off-farm income may serve as additional source of income, farmers are able to purchase larger quantity and quality of foods that serve to increase their dietary diversity. Moreover, an increase in the time taken to trek to the market and increasing cost of transportation to the market decreased household dietary diversity.

#### 3.3.3. Modern food retail market participation impact pathways and dietary diversity

Table 4 reports results of the four potential indirect linkages through which MFRM participation impacts dietary diversity. Our results confirm that the main pathways through which participation in MFRM impacts household dietary diversity are: Income changes, consumption of poultry products produced by the farmers, production of vegetables (for sale and consumption) using poultry dropping as manure, and through changing gender roles within the household. The results indicate that area of vegetable cultivated using poultry dropping, poultry farm income, and value of own poultry products consumed all demonstrated positive and significant effects on farm households’ dietary diversity. Share of poultry revenue controlled by men, which is a proxy used in determining changing gender roles within the households, shows a negative and significant effect on dietary diversity. This implies that female control of poultry farm revenue increased household dietary diversity and this is because women are primarily saddled with the responsibility to provide cooking and provide food for their families.

#### 3.3.4. Modern food retail market participation and individual factor influencing dietary diversity

Table 4 summarizes results of the impact of modern food retail market participation on the main factors (area of vegetable cultivated using poultry dropping, poultry farm income, share of poultry farm revenue controlled by men, and value of own poultry products consumed by the farmers) that influence household dietary diversity. Participation in MFRM significantly increased the area of land cultivated with vegetable using poultry dropping; income from poultry farming, share of poultry farm revenue controlled by men, and consumption of poultry products produced by the farmers. This implies that MFRM participation was positively and significantly (at 1% level) associated with poultry farming income, consumption of poultry products produced by the farmers, area of vegetable cultivated using poultry droppings, and share of poultry farm revenue controlled by men. These variables are the pathways through which MFRM participation impact dietary diversity.

## 4. Discussion

This paper analyzed the MFRM participation by poultry farming households and its impact on their nutritional attainment, as measured by the dietary diversity score, in Southeast Nigeria. In this section, we discussed the findings of the study; MFRM participation, dietary diversity, and linkages. The results indicate that, overall, MFRMs participation is associated with increasing dietary diversity.

The analysis of the dietary diversity of the households shows varying degrees of the number of food groups consumed by poultry farmers that participated in MFRM channels and traditional markets. Farmers participating in MFRM consume about eight food groups while their counterparts who supplied traditional markets consume approximately six food groups. This confirms that farmers participating in MFRM have better access to foods and more diversified diets than those supplying traditional markets. This is similar to the result of the work of Chege et al. [1], where vegetable farmers supplying supermarkets recorded more calorie and micronutrients consumption than farmers under traditional channels. Similarly, Ogutu et al. [42] found higher consumption of calorie and micronutrients in more commercialized farm households than less commercialized households.

Participation in MFRM increased farming households’ dietary diversity. This may not be unconnected with the fact that farmers supplying MFRMs are more likely to sell their poultry products at better prices and earn higher income than those selling at the traditional markets. With increased income, MFRM farmers’ demand and consumption of diverse foods would also increase. Therefore, participation in MFRM supply chains increases poultry farm households’ dietary diversity. Previous studies, see [1,25,45], found similar results. They [1,25,45] found that participation in supermarket channels significantly increased farm households’ nutrition. Therefore, supplying MFRM is likely to increase farmers’ economic access to quality foods and consumption of diversified diets. This forms part of our hypothesis on the various pathways through which participation in MFRM affects dietary diversity of poultry farmers.

The possible pathways through which participation in MFRM channels impact poultry farm households were increased poultry farm income, increased consumption of own poultry products, cultivation of vegetables with poultry dropping, and intra-household changing gender roles. These pathways were tested and participation in MFRM channels demonstrated positive and significant effect on poultry farm income, area of vegetable cultivated using poultry dropping, share of poultry farm revenue controlled by male farmers, as well as value of own poultry products consumed. Supplying MFRM increased poultry farmers’ income by ₦517,872. This is related to previous studies [1,10,22,45,46,47] where supplying supermarkets and other high-value markets resulted in incremental income of smallholder farmers. Gains in income create better access to quality foods and diversified diets for farming households [48]. The income gains result in higher demand for additional and diverse foods [1], which in turn leads to increased dietary diversity. Secondly, the results suggest that poultry farmers participating in MFRM have higher dietary diversity score through increased consumption of self-produced poultry products. More specifically, farmers with contractual agreements with MFRM are usually medium-to-large-scale producers controlling a large number of birds and the products. Entering into contractual agreements with MFRM reduces price volatility and incentivizes farmers to scale and increase productivity [49]. According to Chege et al. [1], any product that does not meet strict contract standards are not delivered to the MFRM channels, rather, they are left for household use or consumption thereby increasing household food access and diversity, especially for MFRM farmers. Thirdly, the results suggest that poultry farmers participating in MFRM have higher dietary diversity score through production of vegetables using poultry dropping as manure. It was observed during the data collection that MFRM farmers consumed more of their own produced vegetables using poultry dropping because of their belief that it is safer and nutritious than vegetables purchased in the open market which they assume may have been produced with inorganic fertilizer. Since organic vegetable farmers (in this case, using poultry dropping as manure) who supply to MFRM may be bound by the two-party supply agreements, there are incentives for farmers to maintain or increase production to meet the terms of the agreements. Increased vegetable production using poultry dropping as manure can lead to increased sales and consumption at home. More specifically, increased vegetable production resulting from using poultry dropping as manure increases farm households’ consumption of vegetables leading to better dietary diversity and quality. Vegetables are rich in vitamin contents, and farmer households benefit from increased vitamin contents in their diets. Lastly, results suggest that poultry farmers participating in MFRM have lower dietary diversity score indirectly through changing gender roles within the household. Men are taking over farming enterprises previously dominated by women especially where commercialization of such enterprises has proven to be more profitable [50,51,52]. The results suggest that any reallocation of control of poultry revenue from female to male will lead to reduction in household dietary diversity [53]. Studies have confirmed that the primary caregiver in Nigerian households are females [1,50,51], hence any reallocation of control of farm revenues from female to male can further disempower the female by lowering their purchasing power and this may result in lower household nutrition and dietary diversity [54]. Women spend more of their income on households’ foods, diet, and nutrition than men [1,50,51].

## 5. Conclusions

This paper analyzed the relationship between poultry farmers participation in modern food retail markets and household dietary diversity. Studies on the nutrition impacts of MFRM participation by poultry farmers in developing countries are rare and this paper aimed at filling this gap. The paper uses cross-sectional survey and appropriate estimation techniques to find evidence of the impact of participation in MFRM on poultry farm households’ dietary diversity. We tested the hypothesis of the possible pathways through which participation in MFRM can impact on dietary diversity of poultry farmers.

Our findings show that MFRM participation improved dietary quality of poultry farm households. There are four pathways identified in this study through which MFRM participation impacts dietary diversity, namely (1) income changes, (2) consumption of poultry products produced by the farmers, (3) production of vegetables (for sale and consumption) using poultry dropping as manure, and (4) changing gender roles within the household.

Increased poultry farm income led to increased household dietary diversity. MFRM-participating poultry farmers benefit from higher incomes and this income gains can be used to purchase additional food and diverse foods. Secondly, consumption of poultry products produced by the farmers is associated with significantly higher dietary diversity. This may stem from factors which cause the poultry farmer to specialize. For example, contractual agreements between a modern food retail market and a farmer can provide price guarantees and incentivize the farmer to specialize in poultry production, thus boosting food productivity and food for the farmer. Similarly, production of vegetables using poultry dropping as manure increases dietary diversity. Increased vegetable production resulting from using poultry dropping as manure can boost farmer incomes through sales, and also increase household consumption. Conversely, the fourth linkage has negative impact on dietary diversity. The result shows that MFRM participation is associated with male control of poultry revenues, and the male household members are associated with less dietary expenditure and diversity.

This paper indicates that food sector modernization and the participation of poultry farmers therein have major implications. These implications are the potential for improved nutritional attainment among smallholder farmers and increasing productivity and income boost in the farm sector of developing countries. The existence of indirect linkages indicates that efforts aimed at better understanding the complex relationship between agriculture, supply chains, and household nutrition is necessary to achieve desired outcomes. The result of the impact of MFRM participation on intra-household gender decisions also calls for attention to be given towards gender equality and female farmers support. These findings are important for developing country policy making since the majority of developing countries’ farmers are smallholder, poor rural farmers. The findings also indicate that policies which encourage food market modernization to encourage rural economic development in Nigeria should be encouraged.

## Figures and Tables

**Table 1:** Socioeconomic characteristics of the respondents.

Variable	Proportion/Mean *	Std. Dev.
Participation in MFRMs (yes = 1, no = 0)	27%	
Gender of household head (male = 1, female = 0)	70%	
Marital status (married =1, not married = 0)	82%	
Number of under-five (count)	1.39	1.42
Household size (count)	5.39	1.91
Number women in reproductive age (count)	1.39	1.14
Education of spouse (years)	7.93	3.27
Education of head of household (years)	10.43	3.47
Poultry farming experience (years)	11.94	8.27
Age of household head (years)	46.93	9.61
Age of spouse (years)	41.37	8.49
Contact with a veterinarian (yes = 1, no = 0)	56%	
Extension contact (yes = 1, no = 0)	34%	
Ownership of television (yes = 1, no = 0)	65%	
Ownership of radio (yes = 1, no = 0)	83%	
Ownership of mobile phone (yes = 1, no = 0)	90%	
Ownership of car (yes = 1, no = 0)	35%	
Access to credit (yes = 1, no = 0)	18%	
Household head is a member of cooperative society (yes = 1, no = 0)	52%	
Spouse is a member of cooperative society (yes = 1, no = 0)	22%	
Time taken to trek to market (min)	37.58	42.80
Cost of transportation to the market (₦)	159.25	137.76
Time taken to trek to the nearest tarred road (min)	13.40	13.75
Off-farm income (₦)	58101.82	76870.00
Area of land cultivated with vegetables using poultry dropping (ha)	0.18	0.20
Male share of revenue from poultry production (male = 1, female = 0)	53%	
Annual poultry farm income (₦)	787054.50	670186.90
Value of own poultry products consumed (₦)	49923.54	70319.92
Potable water access (yes = 1, no = 0)	78%	
Ownership of refrigerator (yes = 1, no = 0)	59%	
Access to electricity (yes = 1, no = 0)	70%	
Sample size	220	

Authors’ calculation. Symbol (*) represents both proportion and mean of variables. Std. Dev. represents standard deviation of the variable from the sample mean. ₦ represents Nigerian Naira. 1 USD ≅ ₦ 305 as at 30 May, 2019.

**Table 2:** Household dietary diversity according to modern food retail market (MFRM) channel.

	Modern Food Retail Market Channel	Traditional Market Channel	Pooled
Dietary Diversity	8.25 (1.54)	5.74 (2.24)	6.43 (2.17)
Score	60	160	220

Values in parenthesis are standard deviations.

**Table 3:** Instrumental variable regression estimates of the impact of MFRM participation on dietary diversity.

Variable	(1) MFRM Participation Equation	(2) Dietary Diversity Equation
Participation in MFRM		1.127 *** (3.13)
Extension contact	0.20 *** (4.60)	
Gender of household head	0.15 *** (3.33)	0.30 *** (2.64)
Marital status	0.14 *** (2.66)	0.06 ** (2.16)
Number of under-five	0.03 ** (2.14)	0.05 *** (2.66)
Household size	−0.006 (−0.54)	−0.002 (−0.03)
Number women in reproductive age	−0.027 (−1.35)	0.04 ** (2.30)
Education of spouse	0.002 *** (3.09)	0.008 ** (2.18)
Education of head of household	0.0002 ** (2.04)	0.01 ** (2.42)
Poultry farming experience	0.008 ** (2.46)	−0.02 (−0.79)
Age of household head	−0.0019 (−0.87)	−0.014 (−1.08)
Age of spouse	−0.005 ** (−2.08)	−0.02 ** (−2.29)
Contact with veterinarian	0.16 *** (4.09)	0.14 (0.42)
Ownership of television	0.01 (0.37)	0.21 * (1.72)
Ownership of radio	0.11 * (1.92)	0.22 ** (2.45)
Ownership of mobile phone	0.25 *** (3.16)	−0.34 (−0.61)
Ownership of car	0.04 * (1.79)	−0.18 (−0.64)
Access to credit	0.43 *** (7.92)	0.34 (0.51)
Member of cooperative society (household head)	0.07* (1.67)	−0.17 (−0.64)
Member of cooperative society (spouse)	0.10 * (1.89)	0.48 * (1.83)
Time taken to trek to market	0.001 * (1.95)	−0.003 * (−1.69)
Cost of transportation to the market	−0.00006 (−0.38)	−0.0018 * (−1.81)
Time taken to trek to the nearest tarred road	−0.0004 (−0.28)	0.002 (0.23)
Off-farm income	3.69 × 10^−07^** (2.39)	1.52 × 10^−06^* (1.86)
Potable water access	−0.06 (−1.13)	−0.32 (−0.91)
Ownership of refrigerator	0.01 * (1.88)	0.38 ** (2.38)
Access to electricity	0.089 *** (2.96)	0.68 * (1.84)
Constant	0.38 ** (1.97)	7.70 *** (6.34)
Sample size (N)	220	

* significant at 10% level; ** significant at 5% level; *** significant at 1% level. Values in parenthesis are *z*-values.

**Table 4:** Impact pathway of MFRM participation on dietary diversity.

Variable	Dietary Diversity
*Impact on dietary diversity*	
Area of vegetable cultivated using poultry dropping (ha)	0.23 ** (2.13)
Share of poultry farm revenue controlled by men (dummy)	−0.29 ** (−2.30)
Poultry farm income (₦)	3.34 × 10^−07^*** (2.70)
Value of own poultry products consumed (₦)	1.29 × 10^−06^** (2.21)
Constant	6.37 *** (5.64)
*Impact on area of vegetable cultivated using poultry dropping (ha)*	
High value market participation (dummy)	0.32 *** (9.57)
Constant	−0.18 * (−1.92)
*Impact on share of poultry production revenue controlled by men* (dummy)	
High value market participation (dummy)	0.40 *** (4.26)
Constant	0.21 * (1.78)
*Impact on poultry farm income (₦)*	
High value market participation (dummy)	517872.40 *** (3.55)
Constant	988624.00 ** (2.48)
*Impact on value of own poultry products consumed (₦)*	
High value market participation (dummy)	30377.79 *** (3.05)
Constant	−14051.18 ** (−2.48)

* significant at 10% level; ** significant at 5% level; *** significant at 1% level. Values in parenthesis are *z*-values.

## References

[1] Chege C.G.K., Andersson C.I.M., Qaim M. (2015). Impacts of Supermarkets on Farm Household Nutrition in Kenya. World Dev..

[2] Reardon T., Timmer C.P. (2014). Five inter-linked transformations in the Asian agrifood economy: Food security implications. Glob. Food Secur..

[3] Lakatos C., Fukui T. (2014). The Liberalization of Retail Services in India. World Dev..

[4] Rischke R., Kimenju S.C., Klasen S., Qaim M. (2015). Supermarkets and food consumption patterns: The case of small towns in Kenya. Food Policy.

[5] Rao E.J.O., Brümmer B., Qaim M. (2012). Farmer Participation in Supermarket Channels, Production Technology, and Efficiency: The Case of Vegetables in Kenya. Am. J. Agric. Econ..

[6] Reardon T., Barrett C.B., Berdegué J.A., Swinnen J.F.M. (2009). Agrifood Industry Transformation and Small Farmers in Developing Countries. World Dev..

[7] Neven D., Odera M.M., Reardon T., Wang H. (2009). Kenyan Supermarkets, Emerging Middle-Class Horticultural Farmers, and Employment Impacts on the Rural Poor. World Dev..

[8] Rao E.J.O., Qaim M. (2011). The Supermarket Revolution and Impacts on Agricultural Labor Markets: Empirical Evidence from Kenya.

[9] Rao E.J.O., Qaim M. (2013). Supermarkets and agricultural labor demand in Kenya: A gendered perspective. Food Policy.

[10] Andersson C.I.M., Chege C.G.K., Rao E.J.O., Qaim M. (2015). Following Up on Smallholder Farmers and Supermarkets in Kenya. Am. J. Agric. Econ..

[11] Umberger W.J., He X., Minot N., Toiba H. (2015). Examining the relationship between the use of supermarkets and over-nutrition in Indonesia. Am. J. Agric. Econ..

[12] Demmler K.M., Ecker O., Qaim M. (2018). Supermarket Shopping and Nutritional Outcomes: A Panel Data Analysis for Urban Kenya. World Dev..

[13] Asfaw A. (2008). Does Supermarket Purchase affect the Dietary Practices of Households? Some Empirical Evidence from Guatemala. Dev Policy Rev..

[14] Asfaw A. (2011). Does Consumption of Processed Foods explain Disparities in the Body Weight of Individuals? The Case of Guatemala. Health Econ..

[15] Kiria C.G. (2015). Impacts and Impact Dynamics of Smallholder Participation in High-Value Markets in Kenya.

[16] Hawkes C. (2008). Dietary Implications of Supermarket Development: A Global Perspective. Dev Policy Rev..

[17] Kelly M., Seubsman S.A., Banwell C., Dixon J., Sleigh A. (2014). Thailand’s Food Retail Transition: Supermarket and Fresh Market Effects on Diet Quality and Health. Brit. Food J..

[18] Gebru K.M., Leung L., Rammelt C., Zoomers A., van Westen G. (2019). Vegetable Business and Smallholders’ Food Security: Empirical Findings from Northern Ethiopia. Sustainability.

[19] Toiba H., Umberger W.J., Minot N. (2015). Diet Transition and Supermarket Shopping Behaviour: Is there a Link?. Bull. Indones. Econ. Stud..

[20] Tessier S., Traissac P., Maire B., Bricas N., Eymard-Duvernay S., El Ati J., Delpeuch F. (2008). Regular Users of Supermarkets in Greater Tunis Have a Slightly Improved Diet Quality. J. Nutr..

[21] Rupa J.A., Umberger W.J., Zeng D. (2019). Does food market modernisation lead to improved dietary diversity and diet quality for urban Vietnamese households?. Aust. J. Agric. Resour. Econ..

[22] Rao E.J.O., Qaim M. (2011). Supermarkets, Farm Household Income, and Poverty: Insights from Kenya. World Dev..

[23] Ismail M., Kavoi M.M., Eric B.K. (2013). Factors influencing the choice of supermarket channel by smallholder vegetable farmer suppliers in Nairobi and Kiambu Counties, Kenya. J. Agric. Econ. Dev..

[24] Louw A., Jordaan D., Ndanga L., Kirsten J.F. (2008). Alternative Marketing Options for Small-Scale Farmers in the Wake of Changing Agri-Food Supply Chains in South Africa. Agrekon.

[25] Ochieng D.O. (2017). Supermarket Contracts, Income, and Changing Diets of Farm households: Panel Data Evidence from Kenya.

[26] Business Day (2015). The Nigerian Retail Report 2014/2015.

[27] United Nations (2017). World Population Prospects. The 2017 Revision: Key Findings and Advance Tables.

[28] Sahel C.P. (2015). An Assessment of the Nigerian Poultry Sector.

[29] Odah E.O., Daikwo S.I., Mbap S.T., Okpanachi U. (2019). Phenotypic Characterization of Local Chickens (Gallus Gallus Domesticus) In Bekwarra Cross River State, Nigeria. JSM Vet. Med. Res..

[30] Abimiku Y.J., Emeka-Okolie W. (2008). Assessment of the Nigerian poultry market chain Assessment of the Nigerian poultry market chain.

[31] Ordinaka C.F., Anayor B.O., Ogbonnaya G.H., Abasi D.S., Ozeh E.M. (2016). A study of Vegetable Farmers’ Decision to Use Poultry Litter in the Southern Region of Nigeria. African J. Agric. Econ. Rural Dev..

[32] Simon E., Philip D.O., Haruna V., Jabil I.Y., Pewan S.B., Haruna I.M. (2016). Analysis of Women Participation in Livestock Production in Mangu Local Government Area of Plateau State, Nigeria. Int. J. Sci. Appl. Res..

[33] Garba J., Yari A.Y., Haruna M., Ibrahim S. (2013). Traditional Poultry Production: The Role of Women in Kaura-Namoda Local Government Nigeria. African J. Agric. Res..

[34] Ahmed A., Mijinyawa M.S., Adamu A.Y., Suleiman A.O. (2011). Small Holder Poultry Management Practices and Constraints among Women Poultry Farmers in Kano, Nigeria. Niger. Vet. J..

[35] Onyeneke R.U., Nwajiuba C.A., Igberi C.O., Amadi M.U., Anosike F.C., Oko-Isu A., Munonye J., Uwadoka C., Adeolu A.I. (2019). Impacts of Caregivers’ Nutrition Knowledge and Food Market Accessibility on Preschool Children’s Dietary Diversity in Remote Communities in Southeast Nigeria. Sustainability.

[36] Vellema W., Desiere S., D’Haese M. (2016). Verifying Validity of the Household Dietary Diversity Score: An Application of Rasch Modeling. Food Nutr. Bull..

[37] Taruvinga A., Muchenje V., Mushunje A. (2013). Determinants of rural household dietary diversity: The case of Amatole and Nyandeni districts, South Africa. Int. J. Dev. Sustain..

[38] Ruel M.T. (2003). Is dietary diversity an indicator of food security or dietary quality? A review of measurement issues and research needs. Food Nutr. Bull..

[39] Swindale A., Bilinsky P. (2006). Household Dietary Diversity Score (HDDS) for Measurement of Household Food Access: Indicator Guide (v.2).

[40] Kennedy G., Ballard T., Dop M. (2011). Guidelines for Measuring Household and Individual Dietary Diversity: Reprint.

[41] Imbens G.W., Wooldridge J.M. (2009). Recent Developments in the Econometrics of Program Evaluation. J. Econ. Lit..

[42] Ogutu S.O., Gödecke T., Qaim M. (2019). Agricultural Commercialisation and Nutrition in Smallholder Farm Households. J. Agric. Econ..

[43] Lemma M., Gebremedhin B., Hoekstra D., Bogale A. (2016). Current Status of Agricultural Extension Services for Market-Oriented Agricultural Development in Ethiopia: Results from a Household Baseline Survey. Afr. Res. Rev..

[44] Ferroni M., Zhou Y. (2012). Achievements and Challenges in Agricultural Extension in India. Glob. J. Emerg. Mark. Econ..

[45] Seng K. (2016). The Effects of Market Participation on Farm Households’ Food Security in Cambodia: An Endogenous Switching Approach.

[46] Van den Broeck G., Maertens M. (2016). Horticultural exports and food security in developing countries. Glob Food Secur..

[47] Maertens M., Minten B., Swinnen J. (2012). Modern food supply chains and development: Evidence from horticulture export sectors in Sub-Saharan Africa. Dev. Policy Rev..

[48] Jones A.D., Shrinivas A., Bezner-Kerr R. (2014). Farm production diversity is associated with greater household dietary diversity in Malawi: Findings from nationally representative data. Food Policy.

[49] Michelson H., Reardon T., Perez F. (2012). Small Farmers and Big Retail: Trade-offs of Supplying Supermarkets in Nicaragua. World Dev..

[50] von Braun J. (1995). Agricultural commercialization: Impacts on income and nutrition and implications for policy. Food Policy.

[51] Fischer E., Qaim M. (2012). Gender, agricultural commercialization, and collective action in Kenya. Food Secur..

[52] Weinberger K., Pasquini M., Kasambula P., Abukutsa-Onyango M.O., Mithöfer D., Waibel H. (2011). Supply chains for indigenous vegetables in urban and peri-Urban areas of uganda and kenya: A gendered perspective. Vegetable Production and Marketing in Africa: Socio-Economic Research.

[53] Sraboni E., Malapit H.J., Quisumbing A.R., Ahmed A.U. (2014). Women’s Empowerment in Agriculture: What Role for Food Security in Bangladesh?. World Dev..

[54] Hoddinott J., Haddad L. (1995). Does Female Income Share Influence Household Expenditures? Evidence from Cote d’Ivoire. Oxf. Bull. Econ. Stat..

